# An Unusual Cause of Failed Tracheal Decannulation—A Case Report

**DOI:** 10.5005/jp-journals-10071-23223

**Published:** 2019-08

**Authors:** Sailaja Kambhampati, K Lavanya

**Affiliations:** 1 Department of Pulmonary Medicine, Maxcure Hospital, Hyderabad, Telangana, India; 2 Department of Pulmonology, Maxcure Hospital, Hyderabad, Telangana, India

**Keywords:** Decannulation, Failure, Membrane, Vocal cords, Weaning

## Abstract

Failure of decannulation may occur due to unexpected upper airway problems. However, the presence of a membrane in between the vocal cords is usually rare. We report a case of a 46-year-old female, who presented with focal seizures and progressed to status epilepticus. She was put on a mechanical ventilator because of hypoxic arrest. As she required prolonged ventilatory support, tracheostomy and gradual weaning from ventilator support to T-piece was done. Following stable hemodynamics, decannulation trial was attempted which failed. Subsequently, bronchoscopy was done to assess the upper airway. It revealed a thick membrane in between the vocal cords. Further examination with an indirect laryngoscope under general anesthesia confirmed the findings, and the membrance was excised. Decannulation was successful the very following day and the patient was discharged with stable hemodynamics.

**How to cite this article:**

Kambhampati S, Lavanya K. An Unusual Cause of Failed Tracheal Decannulation—A Case Report. Indian J Crit Care Med 2019;23(8):378–379.

## INTRODUCTION

A tracheostomy tube is placed for several reasons: to bypass an upper airway obstruction, failure to wean from mechanical ventilation, impaired neurologic status, and inability to handle excessive secretions.^1–3^ The placement of a tracheostomy tube facilitates the transfer of the patient from the intensive care unit to a weaning facility such as a step-down unit or a long-term care hospital.^4^ A tracheostomy may be only a short-term requirement for patients and should be removed as soon as it is no longer needed. Decannulation describes the process of tracheostomy tube removal once the need for the tube has resolved. Advantages of decannulation include improved vocal cord and swallowing function, improved patient comfort and perceived physical appearance. Decannulation failure is defined as reinsertion of an artificial airway within 48-96 hours after tube removal.

## CASE DESCRIPTION

**Fig. 1:**
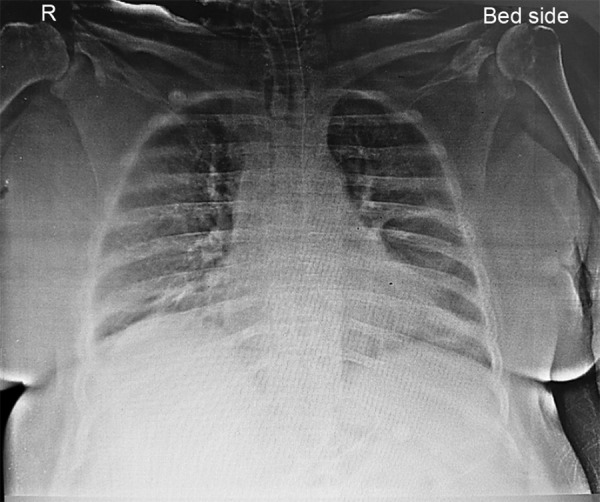
Chest radiograph showing cardiomegaly

A 46-year-old obese, hypertensive, denovo hypothyroid female presented with focal seizures and altered sensorium which progressed to status epilepticus. On assessment, GCS was E4M5V2, pupils were bilaterally reacting to light, hemodynamics were stable, and lungs clear on auscultation. She was being treated with dual antiepileptics, thyroxine, and supportive medication. MRI brain was suggestive of encephalitis, probably of viral or autoimmune etiology (anti-TPO antibodies 901; ANA profile negative; TSH- 9.77). Chest radiograph showed mild cardiomegaly (Fig. 1).

**Fig. 2:**
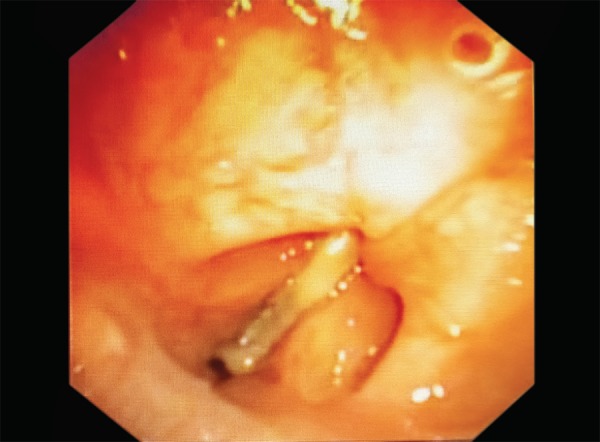
Fixed and adducted vocal cords seen through a bronchoscopy

**Fig. 3:**
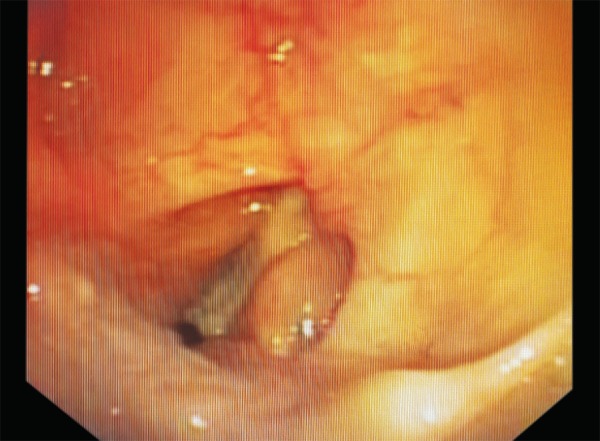
Adducted vocal cords

She was put on a mechanical ventilator three days later because of severe bronchospasm and hypoxic arrest. Five days later, tracheostomy was done as she required prolonged assisted ventilation, tracheostomy was done five days later. Gradual weaning was initiated thereafter. Following stable hemodynamics and resolving primary etiology, decannulation was done. Within minutes, the patient developed severe hypoxia and had to be recannulated immediately. The subsequent attempt, five days later, was a similar failure. Then, video-assisted bronchoscopy was performed to assess the airway. It revealed a white, fixed membrane in between the vocal cords (Figs 2 and 3).

**Fig. 4:**
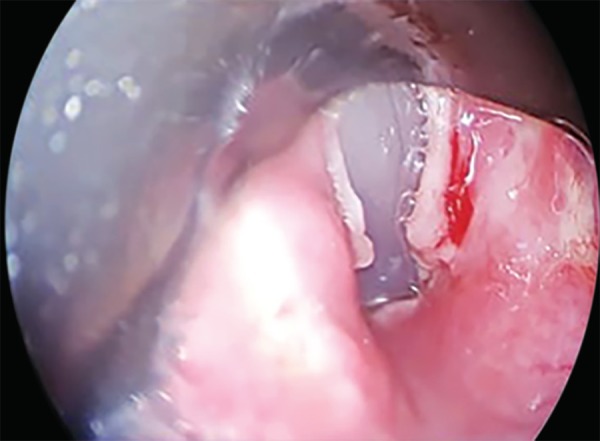
Incised vocal cords showing normal space between the two

Further examination by an otorhinolaryngologist with an indirect laryngoscope proved it to be a membrane, which was promptly excised. The patient was successfully decannulated the very next day and discharged 3 days later with stable hemodynamics (Fig. 4).

## DISCUSSION

Variable degrees of airway obstruction are frequently seen post tracheostomy. The incidence of obstructive lesions in prospective and retrospective examination studies range from 20% and 67% in patients with long-term tracheostomy tubes.^5–7^ Obstruction commonly occurs at the site in contact with cuff, the tip of tube and stoma.^8–10^ It may present as granulations (58%)^6^, tracheomalacia (29-45%)^6,10^ nodulations, polypoids, fibrosis or scarring with constricture or stenosis (14–64%).^5,6,10^ Decannulation is usually well tolerated. Success and failure of decannulation is defined in different ways by different groups. One group defines success as extubation or decannulation and site closure with no consequent respiratory symptoms or blood gas deterioration for at least two weeks and failure is defined as the appearance of respiratory distress and decreases in vital capacity and oxyhemoglobin saturation despite the use of noninvasive IPPV and assisted coughing. Another definition of failure is reinsertion of an artificial airway within 48–96 hours after tracheostomy decannulation. The acceptable decannulation failure rate is reported to range from 2% and 5%.^11^ A systematic approach to patient evaluation, along with judicious use of airway endoscopy, can help identify barriers to decannulation. Fiber optic bronchoscopy allows direct visualization and assessment of the whole upper airway including larynx and trachea above and below the stoma. Assessment of the anatomical, as well as the physiological status of the upper airway, helps in decannulation as well as post decannulation care.

## CONCLUSION

Even though the incidence of decannulation complications may be low,^12^ they can be disastrous and life-threatening. Direct visual inspection with a video-assisted bronchoscope not only enhances the decannulation process but also detects correctable lesions at an early stage. Bronchoscopy is, thus, an invaluable tool in difficult cases of tracheostomy decannulation.
